# Association between gene expression and altered resting-state functional networks in type 2 diabetes

**DOI:** 10.3389/fnagi.2023.1290231

**Published:** 2023-11-29

**Authors:** Yang Zhang, Xin Du, Wen Qin, Yumeng Fu, Zirui Wang, Quan Zhang

**Affiliations:** Department of Medical Imaging and Tianjin Key Laboratory of Functional Imaging, Tianjin Medical University General Hospital, Tianjin, China

**Keywords:** type 2 diabetes, Allen human brain atlas, gene expression, functional connectivity, independent component analysis

## Abstract

**Background:**

Type 2 diabetes (T2DM) is a polygenic metabolic disorder that accelerates brain aging and harms cognitive function. The underlying mechanism of T2DM-related brain functional changes has not been clarified.

**Methods:**

Resting-fMRI data were obtained from 99 T2DM and 109 healthy controls (HCs). Resting-state functional connectivity networks (RSNs) were separated using the Independent Component Analysis (ICA) method, and functional connectivity (FC) differences between T2DM patients and HCs within the RSNs were detected. A partial least squares (PLS) regression was used to test the relation between gene expression from Allen Human Brain Atlas (AHBA) and intergroup FC differences within RSNs. Then the FC differences-related gene sets were enriched to determine the biological processes and pathways related to T2DM brain FC changes.

**Result:**

The T2DM patients showed significantly increased FC in the left middle occipital gyrus (MOG) of the precuneus network (PCUN) and the right MOG / right precuneus of the dorsal attention network (DAN). FC differences within the PCUN were linked with the expression of genes enriched in the potassium channel and TrkB-Rac1 signaling pathways and biological processes related to synaptic function.

**Conclusion:**

This study linked FC and molecular alterations related to T2DM and suggested that the T2DM-related brain FC changes may have a genetic basis. This study hoped to provide a unique perspective to understand the biological substrates of T2DM-related brain changes.

## 1 Introduction

Type 2 diabetes (T2DM) is a polygenic metabolic disorder (Saeedi et al., 2019) that has brought great health and economic burden to individuals and society, which cannot be ignored (American Diabetes Association, 2018). T2DM-related cognitive impairment mainly involves executive and memory functions (McCrimmon et al., 2012; Zilliox et al., 2016). It accelerates brain aging and increases the risk of dementia (Biessels et al., 2006; Mayeda et al., 2015). It has been reported that cognitive function was lower in middle-aged and elderly individuals with T2DM than that without T2DM (Rawlings et al., 2014). So far, the potential mechanism of T2DM-related brain functional changes has not been clarified.

Functional neuroimaging researches have always been committed to revealing the mechanism of T2DM affecting the central neural system. Resting-state functional connectivity networks (RSNs) are involved in cognitive processes (Beckmann et al., 2005). Disruptions in the RSNs are associated with cognitive dysfunction (Gili et al., 2011; Olde Dubbelink et al., 2013). Several RSNs have been identified, and changes of functional connectivity (FC) within networks have been found to be associated with cognitive impairment in T2DM patients (Musen et al., 2012; Chen et al., 2014; Hoogenboom et al., 2014; Lei et al., 2022), including the default mode network (DMN) (Macpherson et al., 2017), the precuneus network, and the executive control network (Wu et al., 2022). From the topological FC network research, patients with early T2DM mostly retained the small-world properties of the brain network while exhibiting abnormal nodal clustering coefficient and characteristic path length in the frontal, temporal lobe, and cingulate gyrus (Zhang D. et al., 2021). These results suggested the reorganization of brain FC networks in T2DM patients (van Bussel et al., 2016). Therefore, RSNs analysis is an effective method for detecting brain functional changes related to T2DM.

T2DM has a strong genetic predisposition (Kwak and Park, 2016). Investigating the link between gene expression and neuroimaging phenotype can help reveal the biological process of T2DM-related brain changes from a unique perspective. Allen Human Brain Map (AHBA) provides spatially matched gene expression data with human brain neuroimaging data, which can help link the brain gene expression with brain FC phenotype. With this approach, neuroimaging-gene expression association studies have been successfully conducted in diseases such as schizophrenia (Morgan et al., 2019; Zong et al., 2022), Parkinson’s disease (Thomas et al., 2021), autism (Romero-Garcia et al., 2019), and Alzheimer’s Disease (Zhang Y. et al., 2021). However, similar studies are limited in T2DM patients.

**Figure 1:**
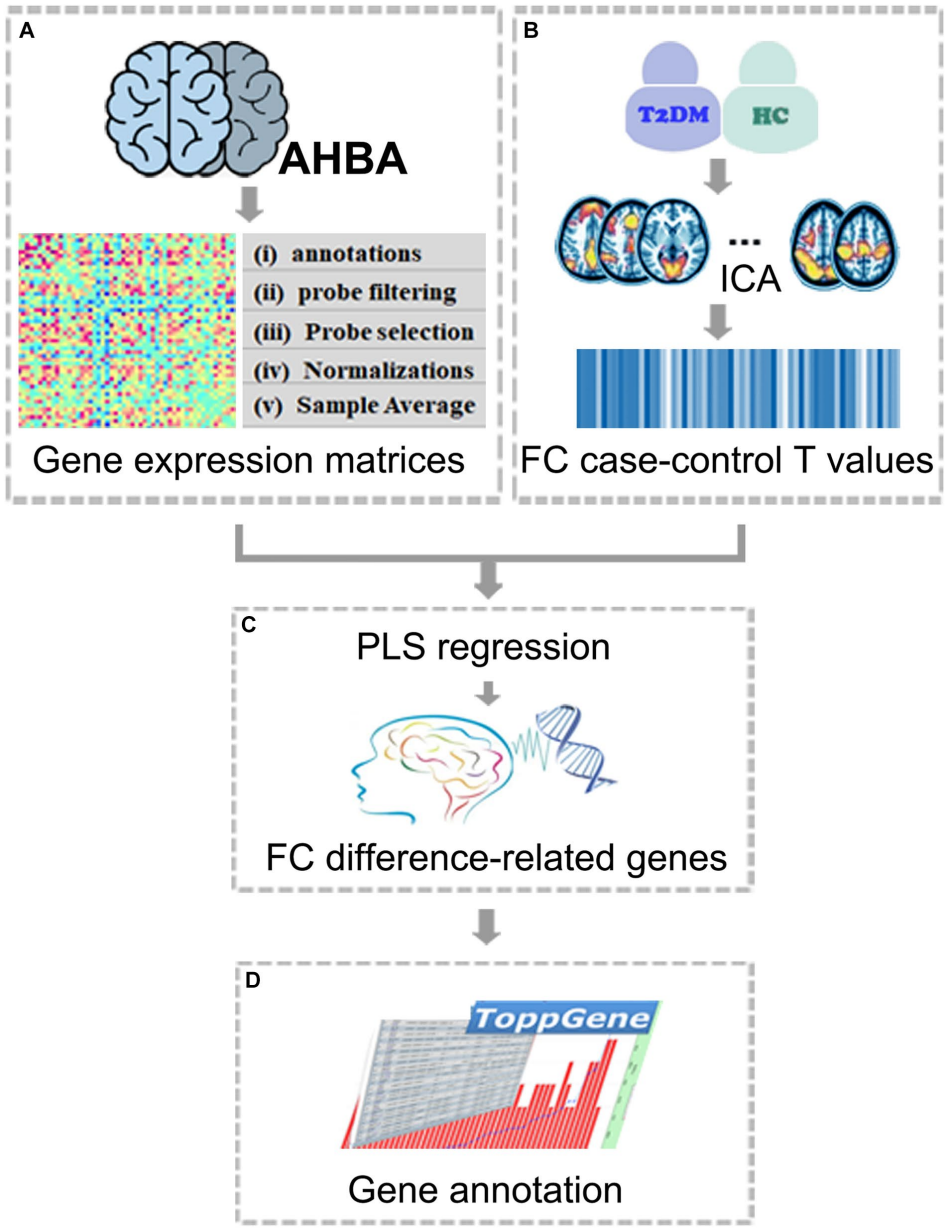
The workflow of the study protocol. **(A)** Acquire the whole-genomic transcriptomic profiles from the AHBA database; **(B)** Obtain the sample-wise gene expression matrix. ICA and component selection were performed on rs-fMRI data. FC case–control T values were obtained by comparing two groups within ICA-identified RSNs; **(C)** Identify FC difference-related genes by investigating correlations between gene expression and FC case–control T values using PLS; **(D)** Functional annotations for FC difference-related genes. AHBA, Allen human brain atlas; FC, functional connectivity; ICA, independent component analysis; PLS, partial least squares.

Independent Component Analysis (ICA) is a frequently used technique for identifying the most prominent RSN in resting-state fMRI (rs fMRI) data (Beckmann and Smith, 2004), which is a data-driven method that automatically explores the temporal correlation between different brain regions in a resting state, detecting and separating multiple RSNs from rs fMRI data (Beckmann et al., 2005). In this study, RSNs were separated by using the ICA and different patterns of internetwork FC in T2DM patients were detected. Then, the FC differences were combined with the gene expression data in AHBA transcriptome data to determine the possible mechanism of T2DM affecting brain function. Partial least squares (PLS) regression was used to test whether FC differences in the RSNs were related to specific gene expression patterns. Finally, those genes whose expression patterns were related to FC differences were enriched and analyzed to determine the biological processes and pathways associated with brain FC changes in T2DM. A workflow of the study protocol was shown in Figure 1. This study conducted an exploratory analysis on the possible mechanism of T2DM affecting brain function and hoped to provide a unique perspective to understand the mechanism of T2DM-related brain changes.

## 2 Materials and methods

### 2.1 Participates

This study was approved by the Ethics Committee of Tianjin Medical University General Hospital. All participants voluntarily provided written informed consent. 109 individuals with T2DM were recruited from the Department of Endocrinology of Tianjin Medical University General Hospital. 119 Well-matched healthy controls (HCs) were recruited from community recruitment. Four T2DM patients and five HCs were excluded due to incomplete scans or cognitive assessment. Subjects with poor blood sample quality (four T2DM patients and five HCs) and poor image quality (two T2DM patients) were also excluded. Finally, 99 individuals with T2DM and 109 well-matched HCs were enrolled. The clinical diagnosis of T2DM was based on the 2010 criteria of the American Diabetes Association (ADA). By screening, patients with retinopathy, nephropathy, and peripheral neuropathy were excluded. As previously described (Ryan and Geckle, 2000), retinopathy was screened by direct ophthalmoscopy, nephropathy by laboratory tests for microalbuminuria, and peripheral neuropathy by clinical examinations. Exclusion criteria were as follows: (i) history of psychiatric disorder; (ii) history of stroke, hemorrhage, trauma, or epilepsy; (iii) alcohol or drug abuse; (iv) family dementia; (v) contraindications for MRI scans.

### 2.2 Clinical data and cognitive assessment

All the participants were right-handed and were in Chinese Hans. The disease course of each T2DM patient was recorded. For all the individuals, education level, height, and weight were recorded and their body mass index (BMI) was calculated. Blood samples were collected in the morning after more than 10 hours of fasting and used for testing fasting blood glucose (FBG) and glycosylated hemoglobin (HbA1c).

Cognitive assessments were conducted before MRI scanning. General cognition was assessed using the MMSE (Folstein et al., 1975). Anxiety and depression were tested using the self-rating anxiety scale (SAS) (Zung, 1971) and the self-rating depressive scale (SDS) (Zung, 1965), respectively. Episodic memory was evaluated by the Rey-Osterrieth Complex Figure Test (ROCF) (Shin et al., 2006) and the Chinese version of the Auditory Verbal Learning Test (AVLT) (Rosenberg et al., 1984).

### 2.3 MRI data acquisition

MRI scans were performed on a 3.0-T MR system (Discovery MR750; General Electric, Milwaukee, WI, United States), using an 8-channel phase array head coil. Blood oxygen level-dependent (BOLD) fMRI data were obtained using a gradient-echo, single-shot, echo-planar imaging sequence. Specific scanning parameters were set as TR = 2000 ms, TE = 45 ms, FA = 90°, FOV = 220 mm × 220 mm, matrix = 64 × 64, slice thickness = 4 mm, slice gap = 0.5 mm, 32 axial slices, 180 volumes. Three-dimensional sagittal T1-weighted images were obtained using a brain volume sequence. Specific parameters were set as TR = 8.2 ms, TE = 3.2 ms, TI = 450 ms, FA = 12°, matrix = 256 × 256, section thickness = 1 mm, 188 continued sagittal sections.

### 2.4 MRI data preprocessing

Statistical Parametric Mapping software (SPM12; www.fil.ion.ucl.ac.uk/spm) embedded in MATLAB was used to preprocess the rs fMRI data. The following steps were performed: (i) remove the first 10 volumes in each time series; (ii) slice timing correction; (iii) motion estimation and correction, subjects with more than 2 mm translational or more than 2° rotational head motion were excluded; (iv) spatially normalization into Montreal Neurological Institute (MNI) space; (v) reslice: 3 mm × 3 mm × 3 mm; (vi) spatially smoothed with a 6-mm full width at a half-maximum Gaussian kernel.

### 2.5 Independent component analysis and component selection

GIFT software^1^ was used to conduct the group spatial ICA analysis. First, 22 ICs were automatically estimated using the minimum description length criteria (Li et al., 2007). Second, a two-step principal component analysis was performed to decompose the fMRI data into 22 principal components. Third, an Informax algorithm (Bell and Sejnowski, 1995) was used to perform the group-level IC estimation. ICA analysis was re-runed 100 times using the ICASSO method to achieve the most stable estimation of IC. Fourth, the subject-level ICs were reconstructed from the group-level ICs using a spatial–temporal algorithm. Finally, z-scores transformation was performed on subject-specific spatial and temporal components to create a normal distribution. By visual observation and according to the resting state network model in the literature (Laird et al., 2011), the components representing noise were excluded, and finally, 13 ICs representing the RSNs were chosen for further analysis. The spatial component was defined as intra-network FC.

### 2.6 Gene expression data processing

Publicly original gene expression data were downloaded from an open-access transcriptomic dataset (Allen Human Brain Atlas (AHBA), http://human.brain-map.org). The AHBA provides transcriptomic data of 20,737 genes taken from 3,702 spatially distinct tissue samples obtained from six postmortem adult brains. Here, the abagen toolbox,^2^ an open-access software package with a standardized workflow (Markello et al., 2021) for transcriptomic data, was conducted to link gene expression and neuroimaging data. The standardized sample-based workflow included four major steps: (i) Update probe-to-gene annotation, the updated probe-to-gene annotations generated by Arnatkeviciute et al. (2019) were used in the workflow; (ii) Intensity-based filtering of probes, we removed the probes which signal that did not exceed 50% above the background noise; (iii) Probe selection, differential stability is adopted as the selection method, that is, the probe with the highest average correlation to other probes across brain regions for every pair of donors was retained when there are more than two probes indexing the same gene; (iv) Normalization, by using the scaled robust sigmoid (SRS) measure, both sample and gene normalization were conducted for each donor to correct the donor effects. After the above steps, we ultimately obtained the expression values of 15,609 genes of the 3,452 brain samples. The sample × gene expression matrix of 3,452 × 15,609 from the six donors was used for further analysis in our study.

### 2.7 Transcription-imaging association

Before gene expression-neuroimaging association analysis, the sample correspondence between gene expression and fMRI data should be established. First, using the MNI coordinate of each tissue sample in the AHBA data as a center, defined a sphere with a radius of 6 mm, which is twice the size of the voxel, to obtain gene expression data. Then extract the average T-statistic value from the FC case–control T-value map. The following correlation analysis will be conducted within ICA-identified RSNs with significant intergroup FC differences. To detect genes whose expression levels were significantly associated with FC difference, PLS regression was used, with gene expression data as the independent variable (X) and the regional FC case–control T-value of RSN as the dependent variable (Y). The PLS regression was performed using 15 components, and percentage variances in Y explained by the 15 components were plotted (Supplementary Figure S1). A 1,000 times spatial permutation test was utilized to correct the PLS regression results. PLS1 and PLS7 survived the permutation test and therefore adopted for the following enrichment analyses. Genes’ weight in the PLS1 and the PLS7 were evaluated using a Bootstrapping method. After Bonferroni correction (*p* < 0.05), gene sets reliably contributed to the PLS1 and the PLS7 were obtained.

### 2.8 Gene enrichment analysis and functional annotation

The FC difference-related genes (the PLS1 and PLS7 gene sets) were put into the Toppgene^3^ to perform enrich analyses for biological processes and pathways. Multiple comparisons were performed using the Benjamini and Hochberg method for false discovery rate (FDR-BH correction, *p* < 0.05).

### 2.9 Statistical analyses

Functional connectivity analyses were conducted by using SPM12 software.^4^ The 13 retained ICs represented different brain RSNs. To identify the spatial distribution pattern of the FC of each RSN, a voxel-wise one-sample T-test was performed on the spatial maps of each RSN in both T2DM patients and HCs. A family-wise error (FWE) correction (*p* < 0.05) was used for multiple comparison corrections. Brain regions exhibiting statistically positive FC in each RSN were binarized. For each RSN, a mask was generated by taking the union of the statistically positive FC regions in T2DM patients and HCs for subsequent statistical analysis.

Using a two-sample T-test, voxel-based intergroup comparisons of FC were performed in 13 RSNs within their respective mask, and age, sex, and education were controlled. Multiple comparison corrections were conducted using an AlphaSim algorithm (*p* < 0.05) (single voxel uncorrected *p* = 0.001, 1,000 simulations, within the mask of each RSN). Regions with significant intergroup differences were defined as regions of interest (ROI). Averaged FC values within each ROI were extracted for subsequent correlation analyses.

The Statistical Package for the Social Sciences (SPSS version 22.0) was used to test the intergroup difference in the clinical and cognitive data. Two-sample *T*-tests were conducted for normally distributed continuous variables, Mann–Whitney U-tests were used for continuous variables with a non-normal distribution, and categorical variables were assessed with a chi-squared test. Partial correlation analyses were performed to test the correlation between the extracted FC value of ROIs and cognitive assessments after controlling for age, education, and gender. The significance level was set as *p* < 0.05.

## 3 Result

### 3.1 Demographics, clinical indicators and cognitive status

The main demographics, cognitive status, and clinical indicators are shown in Table 1. Compared to HCs, the BMI, FBG, and HbA1c were significantly higher in T2DM patients (*p* < 0.001).

**Table 1:** Demographics, clinical data and cognitive assessment.

Characteristics	Type 2 diabetes (*n* = 99)	Healthy controls (*n* = 109)	Statistical value	*p* value
*Demographics*				
Age (years)	60 (41, 76)	59 (42, 74)	−1.182	0.237
Gender (M/F)	53/46	52/57	0.705	0.401
Education (years)	11 (3, 17)	11 (3, 18)	−0.084	0.933
*Clinical Data*				
BMI (kg/m2)	26.13 ± 2.67	24.64 ± 3.03	3.741	< 0.001^*^
BP (hypertension /normal)	50 / 49	45 / 64	1.778	0.182
FBG (mmol/L)	7.14 (3.57, 16.87)	5.38 (4.52, 9.91)	−8.633	< 0.001^*^
HbA1c (%)	7.00 (5.40, 12.80)	5.60 (4.82, 7.50)	−10.067	< 0.001^*^
HbA1c (mmol/mol)	53.01 (35.52, 116.39)	37.71 (29.18, 58.47)	−10.067	< 0.001^*^
*Cognitive assessment*				
MMSE	28 (22, 30)	28 (21, 30)	−0.105	0.916
AVLT Short-term Memory	41.22 ± 8.45	44.10 ± 10.05	−2.225	0.027^*^
AVLT Long-term Memory	8 (0, 14)	9 (0, 15)	−2.418	0.016^*^
ROCF Copy	31 (13, 36)	31 (21, 36)	−0.398	0.691
ROCF Immediate Recall	0.57 (0.15, 0.92)	0.64 (0.04, 0.90)	−0.741	0.082
ROCF Delayed Recall	0.55 (0.11, 0.93)	0.65 (0.00, 1.00)	−2.972	0.003^*^

Two-sample *t*-tests for normalized data, Mann–Whitney U-tests for non-normalized data, and Pearson’s Chi-square test for gender and BP. ^*^p < 0.05 indicates statistically significant differences. AVLT, Auditory Verbal Learning Test; BMI, body mass index; BP, blood pressure; FBG, fasting blood glucose; MMSE, mini-mental state examination; ROCF, Rey-Osterrieth Complex Figure Test; TC, total cholesterol.

The short-term (*z* = −2.225, *p* = 0.027) and long-term (*z* = −2.418, *p* = 0.016) memory of AVLT and delayed recall score of ROCF (*z* = −2.972, *p* = 0.003) were lower in the T2DM patients compared with the HCs.

### 3.2 WM network spatial patterns

**Figure 2:**
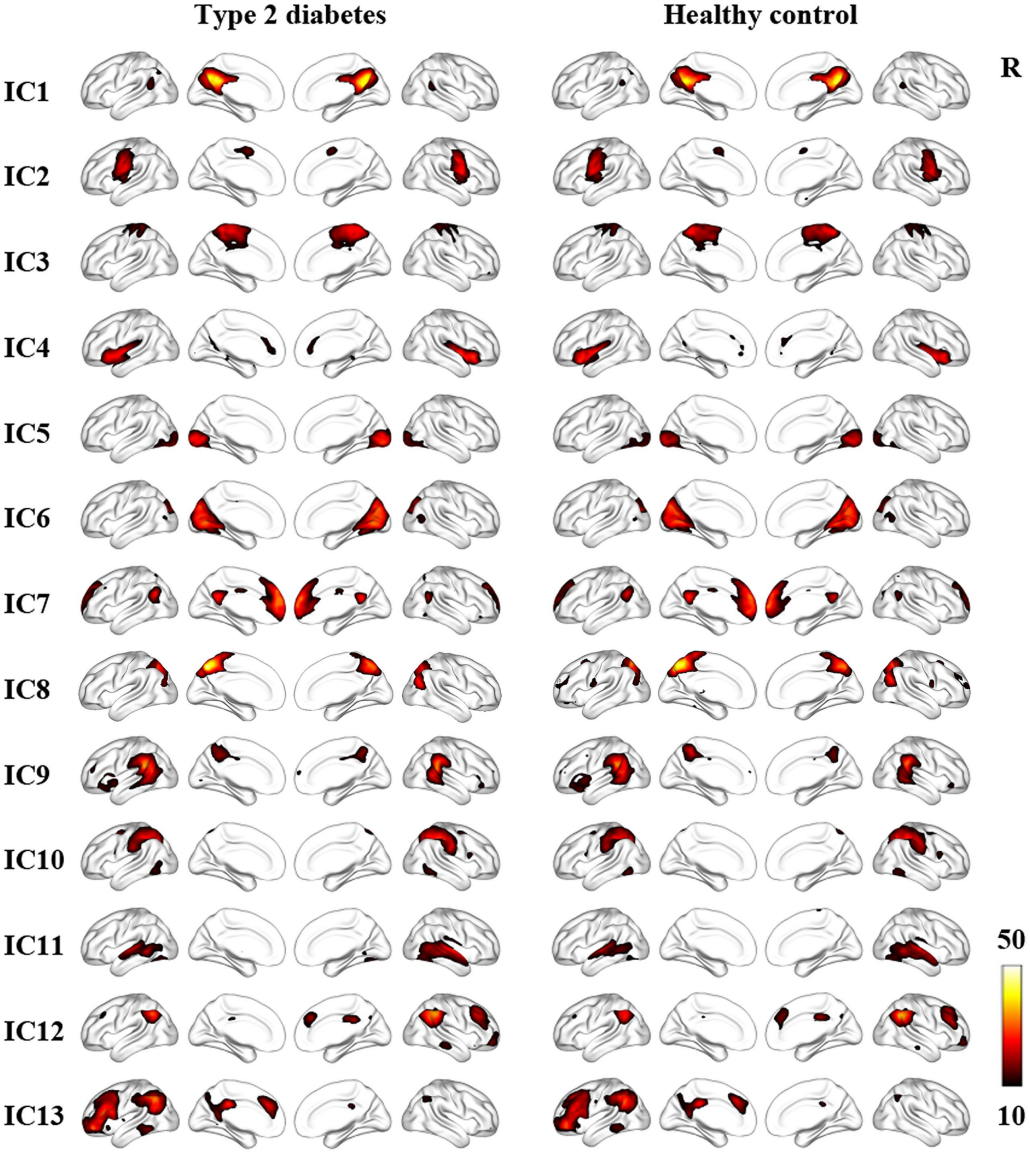
Resting state networks in T2DM patients and HCs identified by ICA. The color bar represents the *t* value by one-sample *t*-test (*p* < 0.05, FWE correction). IC, independent component; R, right.

Thirteen noise-free ICs included the posterior default mode network (pDMN) (IC 1), sensorimotor Network (SMN) (IC 2 and 3), salience network (SAN) (IC 4), visual network (VN) (IC 5 and 6), anterior DMN (aDMN) (IC 7), precuneus network (PCUN) (IC 8), ventral attention network (VAN) (IC 9), dorsal attention network (DAN) (IC10), auditory network (AUN) (IC 11), right frontoparietal network, (RFPN) (IC 12), left frontoparietal network (LFPN) (IC 13). Specific IC maps for the T2DM group and HCs are shown in Figure 2.

### 3.3 Functional connectivity analyses

**Figure 3:**
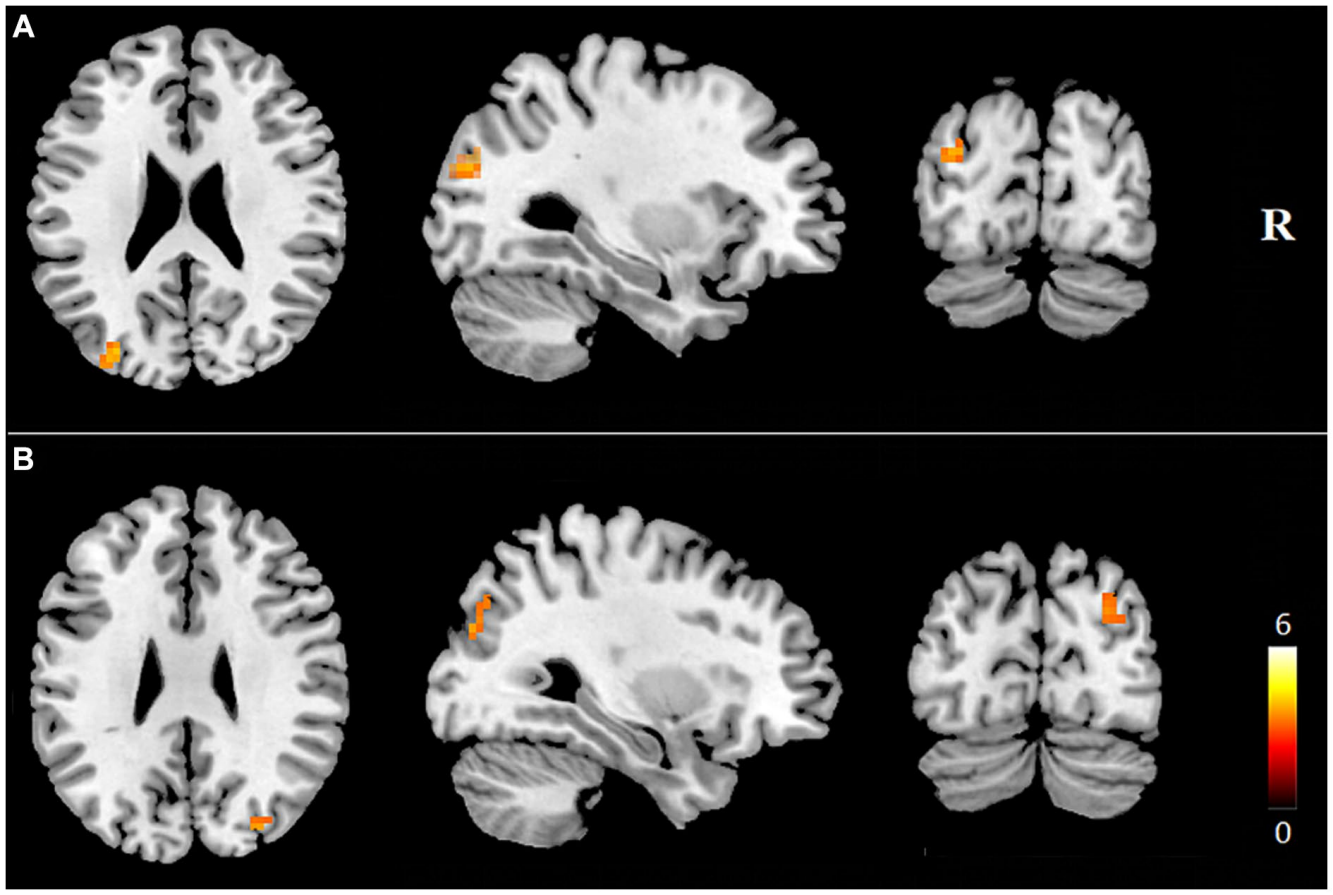
Brain regions showing increased internal network FC in T2DM patients (*p* < 0.05, AlphaSim correction). Brain regions with increased FCs within the precuneus network **(A)** and the DAN **(B)** in the T2DM patients were shown. FC, functional connectivity; R, right.

**Figure 4:**
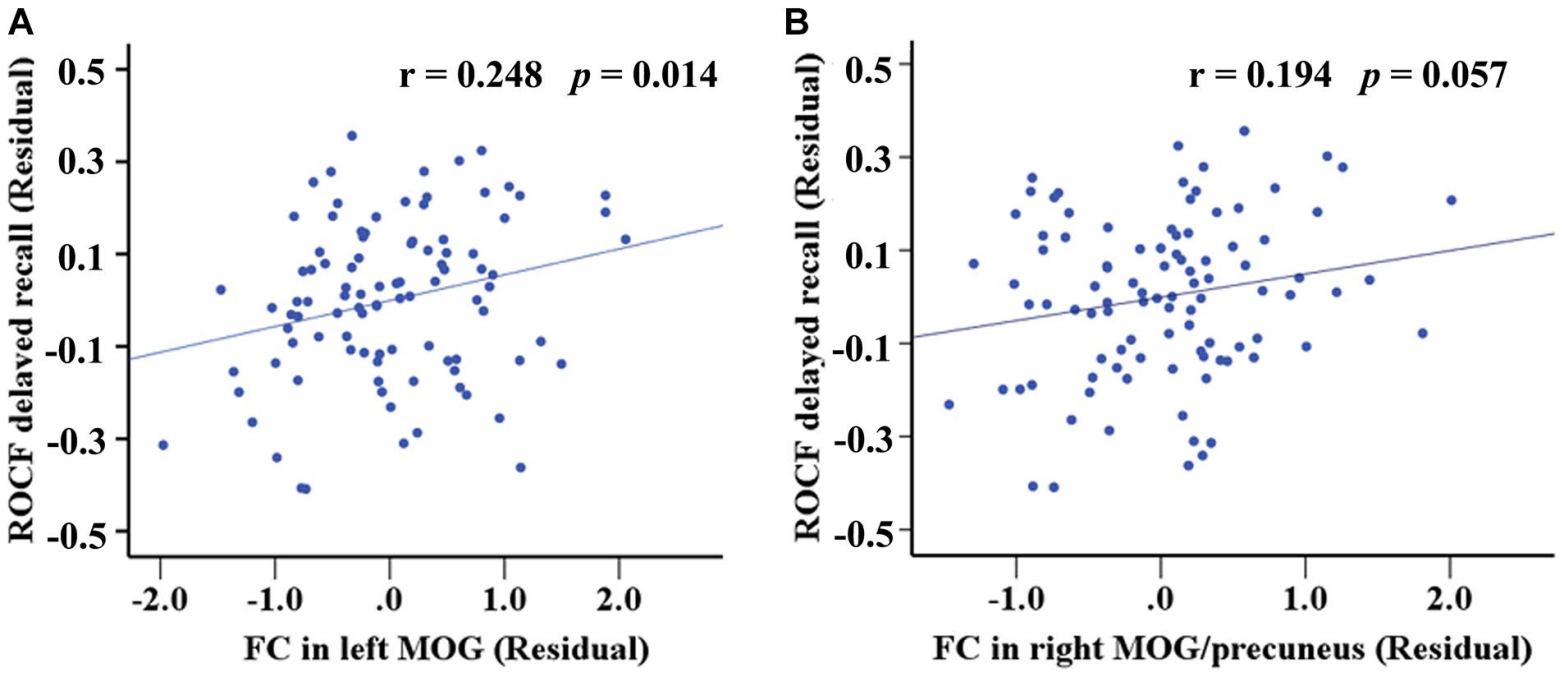
Correlation between the FC differences and the delayed recall scores of ROCF within both PCUN and DAN. **(A)** The FC value in the left MOG of the PCUN was positively correlated with the delayed recall scores of ROCF (*r* = 0.248, *p* = 0.014). **(B)** The FC value in the right MOG / right precuneus of the DAN and the delayed recall scores of ROCF also showed a positive correlation trend (*r* = 0.194, *p* = 0.057).

Internal network FC significantly differed in the PCUN and DAN (*p* < 0.05, AlphaSim corrected). The T2DM patients exhibited significantly increased FC in the left middle occipital gyrus (MOG) of the PCUN (x = −30, y = −81, z = 24, cluster size = 22 voxels, *p* < 0.05, AlphaSim corrected), and the right MOG / right precuneus of the DAN (x = 36, y = −72, z = 42, cluster size = 27 voxels, *p* < 0.05, AlphaSim corrected) (Table 2 and Figure 3). In the T2DM patients, the FC value in the left MOG of the PCUN was positively correlated with the delayed recall scores of ROCF (*r* = 0.248, *p* = 0.014). The FC value in the right MOG / right precuneus of the DAN and the delayed recall scores of ROCF also showed a positive correlation trend (r = 0.194, *p* = 0.057) (Figure 4).

**Table 2:** Brain regions with significantly increased FC values in the T2DM patients compared with the HCs.

RSN	Brain regions	Peak MNI coordinates			Voxels	Peak T value	*p*
		X	Y	Z			
precuneus network	MOG (L)	−30	−81	24	22	3.94	0.042^*^
DAN	MOG/precuneus (R)	36	−72	42	27	4.23	0.041^*^

^*^AlphaSim corrected, *p* < 0.05. DAN, dorsal attention network L, left; MNI, Montreal Neurological Institute, MOG, middle occipital gyrus; R, right.

### 3.4 Associations between gene expression and between group FC difference

**Figure 5:**
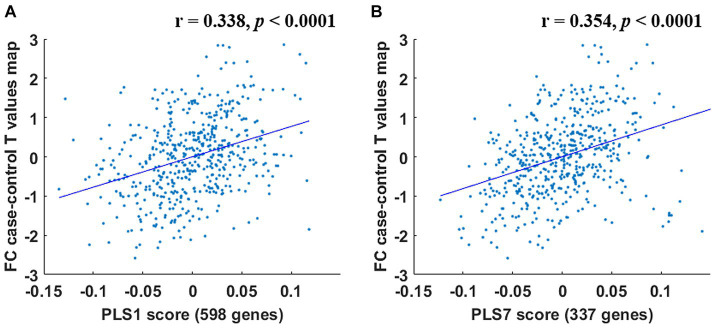
Correlations between the PLS1 and PLS7 components and the intergroup FC difference. PLS1 **(A)** and PLS7 **(B)** components were significantly related to the intergroup FC difference (*p* < 0.0001).

Within the PCUN (IC 8), 598 genes were detected to make significant contributions to the PLS1 (Bonferroni correction, *p* < 0.05) (Supplementary Table S1); 337 genes were identified to make significant contributions to the PLS7 (Bonferroni correction, *p* < 0.05) (Supplementary Table S1). These 2 sets of FC difference-related genes were defined as the PLS1 gene set and the PLS7 gene set, respectively, and were used for subsequent gene functional annotation analyses. Significant PLS components were significantly correlated to the intergroup FC difference (*p* < 0.0001) (Figure 5).

There were no significant PLS components associated with the FC case–control T map within the DAN (IC 10).

### 3.5 Enrichment analysis

**Figure 6:**
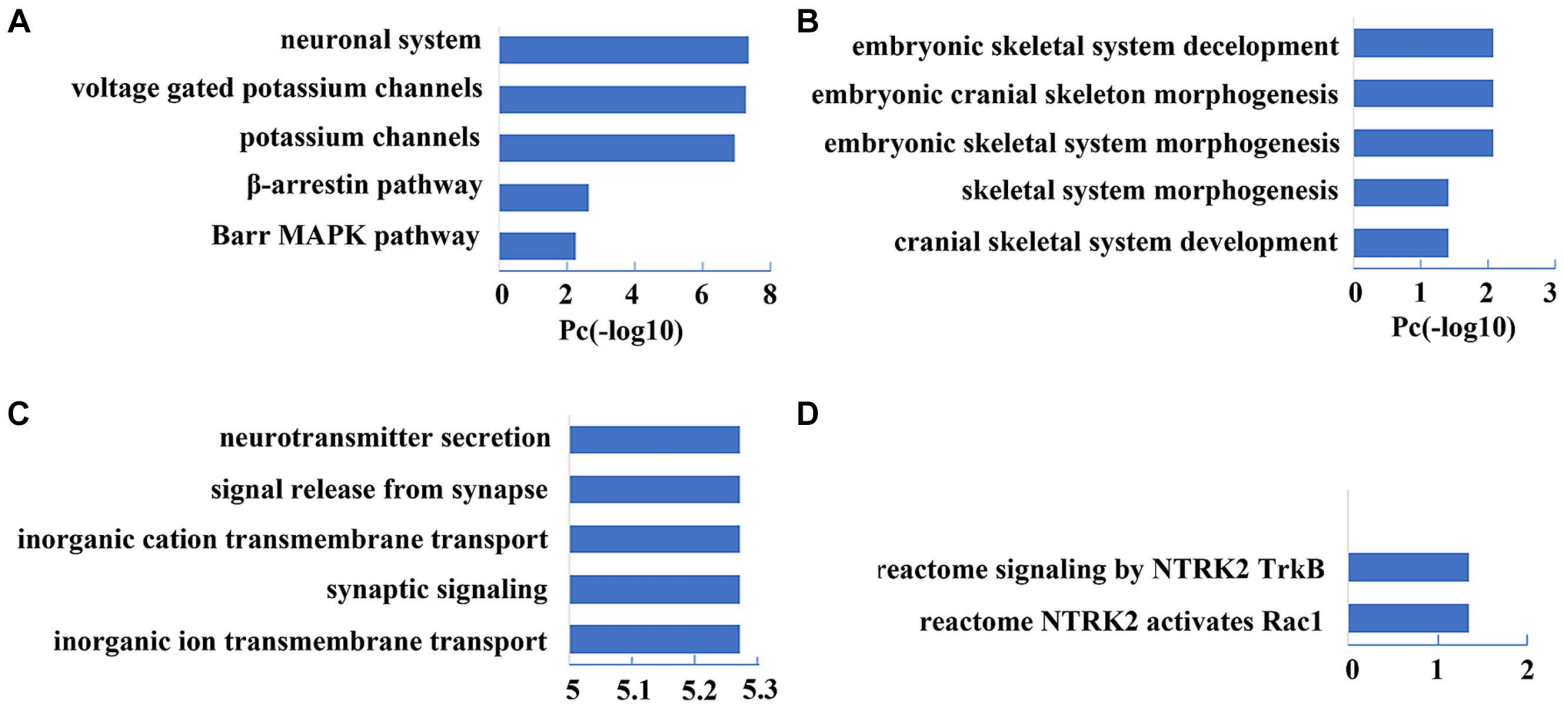
Top five enrichment results of PLS1 and PLS7 gene sets. **(A,B)** Show the pathway and biological process enrichment results of the PLS1 Gene Set (*p* < 0.001, FDR-BH corrected). **(C,D)** Show the pathway and biological process enrichment results of the PLS7 Gene Set (*p* < 0.001, FDR-BH corrected).

The PLS1 gene set showed significant enrichment in neuron-specific terms, including pathways of the neuronal system and potassium channels (FDR-BH correction, *p* < 0.001) and biological processes of neurotransmitter secretion, synaptic signaling, and ion transmembrane transport (FDR-BH correction, *p* < 0.001). The PLS7 gene set exhibited prominent enrichment in the pathway of TrkB-Rac1 signaling (FDR-BH correction, *p* < 0.001); and biological processes of skeletal system morphogenesis/development (FDR-BH correction, *p* < 0.001). The top 5 enrichment results are shown in Figure 6. For detailed information, please see Supplementary Tables S2–S5.

## 4 Discussion

This study carried out an ICA-based internal network FC comparison between the T2DM patients and the HCs. We found that the T2DM patients exhibited increased brain FC in the PCUN and the DAN compared with the HCs, with the main differences in the MOG and the precuneus. PLS regression analysis showed that two genetic components (PLS1 and PLS7) significantly explained the variance of the T2DM-related FC difference within the PCUN. The PLS1 gene set was mainly enriched for the pathway of the neuronal system and potassium channels, and biological processes of neurotransmitter secretion, synaptic signaling, and ion transmembrane transport. The PLS7 gene set was mainly enriched for the pathway of TrkB-Rac1 signaling and biological processes of skeletal system morphogenesis/development.

Both the MOG (Wu et al., 2022) and the precuneus (Cavanna and Trimble, 2006) are crucial for visual cognition-related visual information processing and were commonly reported with alterations in T2DM (Cui et al., 2022). The MOG is located in the primary visual cortex, which is a transit station for visual signals, receiving visual signals from the retina and transferring visual information to the higher visual cortex (Zhen et al., 2018). The precuneus is a major association area integrating higher-order cognitive functions, which has extensive connections with other brain areas and plays a critical role in visual–spatial images and episodic memory (Cavanna and Trimble, 2006). Retinopathy is a common diabetes complication, which can lead to visual impairment. Most studies on T2DM patients with optic nerve-related complications have reported negative alterations in the MOG and the precuneus, including decreased synchronous neural activities, decreased dynamic cerebral activity (Yang et al., 2022), lower degree centrality (Huang et al., 2021), and decreased connectivity (Zhang et al., 2020; Wan et al., 2021). When extended to a wider population of diabetes, the results are inconsistent. Xia et al. (2013) and Li et al. (2021) reported decreased spontaneous neural activities in the MOG, while Liu et al. (2020) reported an increase in spontaneous neural activity in this area. In addition, increased nodal characteristics (Xiong et al., 2020) and functional connectivity (Liu et al., 2020) in the MOG were also reported in T2DM. As for the precuneus, several studies observed compensatory increased FC in the precuneus in T2DM patients (Feng et al., 2021; Lin et al., 2022). In a recent dynamic network connectivity research, the precuneus network was found to be more active in T2DM patients without cognitive decline, and the FC in the left precuneus was positively associated with the long-term memory score of AVLT (Wu et al., 2022). In our study, T2DM patients demonstrated increased FC within the precuneus network in the left MOG and increased FC within the DAN network in the right MOG/precuneus. Compared with those cases with T2DM-related visual system damage, our subjects had no complications or severe cognitive impairment. We speculated that they were still in a compensatory stage where a basic cognitive function can be maintained through a compensatory mechanism of brain functional reorganization. This can be supported to a certain extent by our result of a positive correlation between the FC value in the left MOG of the PCUN and the delayed recall scores of ROCF, as well as the positive correlation trend between the right MOG / right precuneus of the DAN and the delayed recall scores of ROCF in T2DM patients.

During the further enrichment analyses on the two gene sets PLS1 and PLS7, which were closely related to the FC differences within the PCUN, The PLS1 gene set was most enriched for neuronal system and potassium channels, especially voltage-gated potassium channels. The potassium channels are the most broadly distributed and diverse channel in the brain (Abbott, 2020; Kefauver et al., 2020). Among them, the Voltage-gated potassium channels are the largest class of potassium channel families, widely expressed in the central nervous system and involved in a broad range of biological processes, including neuronal excitability generation and transmission, neurotransmitters release, cell proliferation, degradation, and death (Yan et al., 2019). Dysfunctions of potassium channels were found to be related to neurodegenerative diseases (Subramaniam et al., 2014; Rangaraju et al., 2015; Boscia et al., 2017; Yin et al., 2017). For example, finely regulation of potassium channels and their associated proteins plays a critical role in the early onset of Alzheimer’s disease (AD) (Palop et al., 2007; Frazzini et al., 2016). Evidence shows that the amyloid-β (Aβ), a neuropathological hallmark of AD, was also a regulatory factor for potassium channel activity (Plant et al., 2006).

Potassium channels in the nervous system are also important regulators for excitatory synaptic transmission and plasticity and control neurotransmitter release throughout the nervous system. Normal synaptic function is the foundation of brain connectivity, and changes in brain connectivity are related to many neurological diseases. Neurotransmission, brain network dysfunction, and synaptic loss are all involved in neurodegenerative pathogenesis (Reddy and Reddy, 2017). In addition, some mutations in genes encoding voltage gate channel potassium are associated with epilepsy, autism, schizophrenia, and developmental disorders (Baculis et al., 2020).

PLS1 is enriched in potassium channel-related pathways and many biological processes related to synaptic function, indicating the biological substrates underlying T2DM-related FC changes within the PCUN.

The PLS7 gene set was enriched for other pathways of NTRK2 activates RAC1 and signaling by NTRK2/TrkB. Tropomycin receptor kinase B (TrkB) (also known as NTRK2) is a member of the neurotrophin receptor tyrosine kinase (NTRK) family. Neurotrophins bind to the TrkB receptors and activate different downstream signal cascades (Lewin and Barde, 1996). Brain-derived neurotrophic factor (BDNF) is an important receptor of TrkB. BDNF–TrkB signal transduction plays an important role in the development and adult nervous system. Ras-related C3 botulinum toxin substrate 1 (Rac1) is a member of Rho-family GTPases. Rac1 signaling pathway can be activated by the binding of BDNF and TrkB and is involved in regulating actin cytoskeleton, as well as morphogenesis, polarity, migration, axonal growth and guidance, fine and plasticity of dendrites, and synaptic formation of neurons (Luo, 2000; Dickson, 2001; Nikolic and Chernoff, 2002; Sin et al., 2002). Rac1 Activation is necessary for the learning and memory process. Some studies have demonstrated that the Rac1 inactivation will lead to synaptic plasticity changes and memory impairment (Haditsch et al., 2009; Martinez and Tejada-Simon, 2011). Therefore, the pathway enrichment results of the PLS7 gene set may suggest that the TrkB-Rac1 pathway may become another possible biological substrate for T2DM-related FC changes within the PCUN.

Our enrichment analysis of the PLS7 gene set also indicated enrichment in several biological processes related to skeleton morphology and development. The TrkB-Rac1 signaling pathway simultaneously regulates proper synaptic connections and spinal morphogenesis. Therefore, it is unsurprising that genes related to the TrkB-Rac1 signaling pathway were also enriched in biological processes related to skeleton morphology and development. In addition, it has long been recognized that throughout the entire developmental process, the development of nerves and bones is closely intertwined (Marcucio et al., 2011; Richtsmeier and Flaherty, 2013; Gondré-Lewis et al., 2015).

Several limitations should be noted. First, the gene expression data and neuroimaging data were obtained from different participants. However, the overall gene expression of the human population is highly conserved (Stranger et al., 2007), thus the FC difference-related genes obtained in this study should be considered highly conserved among subjects. Second, T2DM patients require long-term medication treatment, and treatment-related effects were inevitable.

In summary, this study linked FC and molecular alterations relevant to T2DM, suggesting that the T2DM-related brain FC changes may have a genetic basis. This study hoped to provide a unique perspective to understand the biological substrates of T2DM-related brain changes.

## Data availability statement

The MRI datasets generated for this study can be found in https://doi.org/10.6084/m9.figshare.24099345. Publicly original gene expression data were downloaded from AHBA (http://human.brain-map.org).

## Ethics statement

The studies involving humans were approved by the Ethics Committee of Tianjin Medical University General Hospital. The studies were conducted in accordance with the local legislation and institutional requirements. The participants provided their written informed consent to participate in this study.

## Author contributions

YZ: Investigation, Writing – review & editing, Data curation, Formal analysis, Methodology, Writing – original draft. XD: Data curation, Formal analysis, Investigation, Methodology, Writing – original draft, Software. WQ: Methodology, Software, Writing – original draft. YF: Writing – original draft, Data curation. ZW: Data curation, Writing – original draft. QZ: Funding acquisition, Investigation, Project administration, Resources, Supervision, Writing – review & editing.

## Footnotes

^1^ http://icatb.sourceforge.net/

^2^ https://abagen.readthedocs.io/en/stable/index.html

^3^ https://toppgene.cchmc.org/

^4^ https://www.fil.ion.ucl.ac.uk/spm/software/spm12
