# Supplementary material for: Association between gene expression and altered resting-state functional networks in type 2 diabetes
Source: Front Aging Neurosci. 2023 Nov 29;15:1290231. doi: 10.3389/fnagi.2023.1290231 (PMC10716229; doi:10.3389/fnagi.2023.1290231)

**Figure S1. Percentage variance explained by the 15 components.** PLS1 and PLS7 survived the 1,000 times spatial permutation test, and were selected for further analyses.


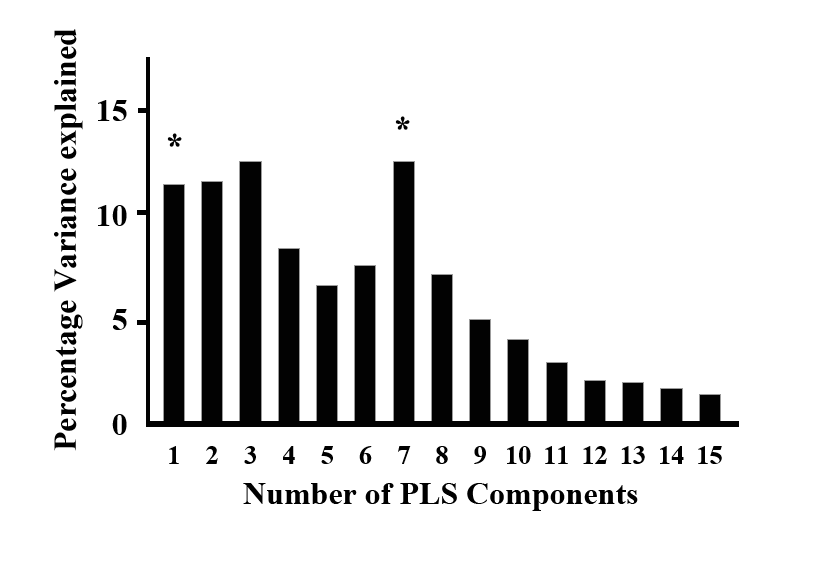

Supplement: Supplementary file 1 [file Table_1.DOCX]
